# Early Adversity and Accelerated Brain Aging: A Mini-Review

**DOI:** 10.3389/fnmol.2022.822917

**Published:** 2022-03-22

**Authors:** Pratik R. Chaudhari, Aastha Singla, Vidita A. Vaidya

**Affiliations:** Department of Biological Sciences, Tata Institute of Fundamental Research, Mumbai, India

**Keywords:** maternal separation, early stress, hippocampus, proteostasis, mitochondria, neuronal survival, neuroinflammation, cognition

## Abstract

Early adversity is an important risk factor that influences brain aging. Diverse animal models of early adversity, including gestational stress and postnatal paradigms disrupting dam-pup interactions evoke not only persistent neuroendocrine dysfunction and anxio-depressive behaviors, but also perturb the trajectory of healthy brain aging. The process of brain aging is thought to involve hallmark features such as mitochondrial dysfunction and oxidative stress, evoking impairments in neuronal bioenergetics. Furthermore, brain aging is associated with disrupted proteostasis, progressively defective epigenetic and DNA repair mechanisms, the build-up of neuroinflammatory states, thus cumulatively driving cellular senescence, neuronal and cognitive decline. Early adversity is hypothesized to evoke an “allostatic load” *via* an influence on several of the key physiological processes that define the trajectory of healthy brain aging. In this review we discuss the evidence that animal models of early adversity impinge on fundamental mechanisms of brain aging, setting up a substratum that can accelerate and compromise the time-line and nature of brain aging, and increase risk for aging-associated neuropathologies.

## Introduction

Early adversity is a potent risk factor for adult psychopathology (Gee, 2021; Teicher et al., 2021). Early stressors such as physical, sexual and emotional abuse, parental neglect/loss, parental/caregiver substance abuse and incarceration disrupt physiological and psychological functioning driving maladaptive health outcomes (Brown et al., 2009; Brenhouse et al., 2019). Animal models attempt to capture the molecular, cellular, neuroendocrine, structural, functional and behavioral changes that arise due to early stress, to gain a mechanistic insight into how early adversity programs psychiatric vulnerability (Tzanoulinou and Sandi, 2017; Blaisdell et al., 2019; Torres-Berrío et al., 2019). While the impact of early stress is experienced by multiple physiological systems, the brain remains the central player as a target of stress and in the top-down control over stress-response pathways (McEwen, 2007). Prior reviews have discussed the influence of early stress on anxio-depressive behaviors and disrupted cognition, accompanied by transcriptional, cytoarchitectural, neuroendocrine and functional changes in diverse limbic brain regions (Chen and Baram, 2016; Teicher et al., 2016; Pervanidou and Chrousos, 2018). Amongst the hallmark features of early stress is that it evokes enduring consequences (Miller et al., 2011; Szyf, 2019). Early adversity exacerbates aging-induced telomere erosion, establishing a pathophysiological basis for enhanced morbidity and mortality (Epel and Prather, 2018; Colich et al., 2020). Clinical literature also indicates that individuals exposed to early stress are more likely to suffer a premature death (Brown et al., 2009). In this review, we critically discuss the evidence that early stress accelerates brain aging.

## Animal Models of Early Adversity

**FIGURE 1:**
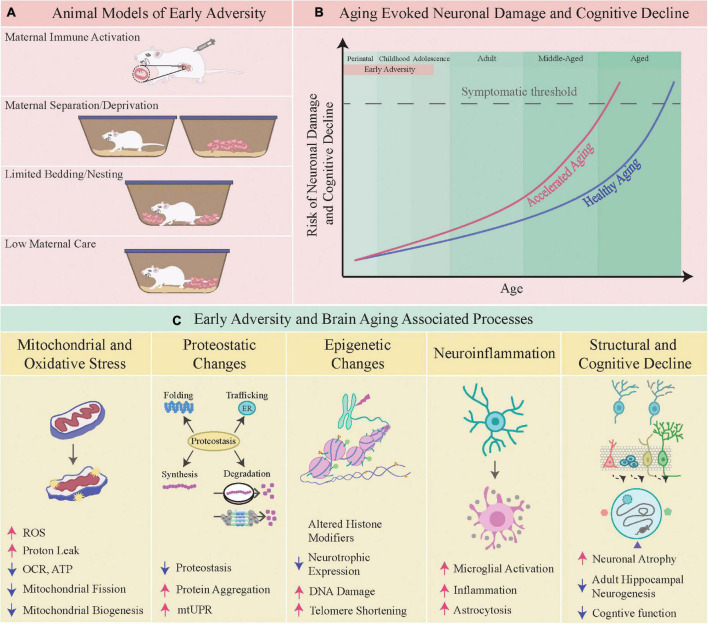
Early adversity and brain aging-associated processes. **(A)** Shown here are specific animal models of early adversity viz. maternal immune activation, maternal separation/deprivation, limited bedding and nesting, and low maternal care. **(B)** The schematic depicts the hastening of brain aging and an earlier onset of neuronal damage and cognitive decline following early adversity. **(C)** Depicted below are some of the aging-associated processes that are perturbed by early adversity, namely the following physiological mechanisms viz. mitochondrial homeostasis, proteostasis, epigenetics, neuroinflammation, structural and cognitive function.

Animal models of early adversity involve stress exposure *in utero*, or during early postnatal time-windows, which can evoke persistent alterations in mood-related behavior, noted long after the cessation of stress (Figure 1A; Schmidt et al., 2011). Gestational stress which involves administration of chronic stress to the dam, or exposure to an inflammatory milieu *in utero*, such as in the maternal immune activation (MIA) model, results in persistent changes in anxio-depressive behaviors in the progeny (Brown and Conway, 2019). During the postnatal temporal window, most rodent models of early adversity capitalize on perturbation of dam-pup interactions and fragmented caregiving behavior from the dam (Orso et al., 2019). These include perturbed licking, grooming, and arched back nursing behavior (LGABN), maternal deprivation (MD), maternal separation (MS), maternal separation combined with unpredictable stress to the dam (MSUS), or limited access to bedding and nesting (LBN) (Caldji et al., 2000; Ladd et al., 2000; Molet et al., 2014; Walker et al., 2017). Common across these models are enhanced anxio-depressive behaviors in the progeny, often accompanied by perturbed cognitive, reward and social behavior (Syed and Nemeroff, 2017; Tzanoulinou and Sandi, 2017; Birnie et al., 2020). Juvenile stress models are usually initiated post-weaning from the dam during the peripubertal window, and for the purposes of this review we have restricted our discussion to early adversity models that involve time-windows prior to juvenile life. Early adversity disrupts stress-responsive neuroendocrine pathways, drives neuroinflammatory states, evokes epigenetic changes, and results in structural and functional changes in neurocircuits that regulate anxio-depressive behaviors, including the hippocampus, prefrontal cortex (PFC), amygdala, and the brain-stem monoaminergic nuclei (Babenko et al., 2015; Teicher et al., 2016; Agorastos et al., 2019; Brenhouse et al., 2019). In this review, we discuss the evidence of an altered brain aging trajectory as a consequence of early adversity, focusing predominantly on studies from animal models.

## Hallmark Signatures of Brain Aging

Aging is characterized by a time-dependent loss of molecular, cellular, structural and functional integrity leading to impaired homeostasis (López-Otín et al., 2013). Accompanying the aging-evoked attrition in all organ systems, “brain aging” also exhibits hallmark features with steady and cumulative decrements noted in structure and function, spanning from atrophy-associated cognitive decline to motor deficits (Mattson and Arumugam, 2018; Oschwald et al., 2019). The characteristic signatures of “brain aging” include mitochondrial dysfunction, oxidative stress, compromised neuronal bioenergetics, impaired proteostasis, perturbed DNA repair, altered intracellular signaling, and a cumulative buildup of neuroinflammatory states (Mattson and Arumugam, 2018). Distinct brain regions show individual variation in the extent of their vulnerability to aging-associated neuronal loss, with the hippocampus, cerebral cortex and cerebellum reported to exhibit both synaptic and cellular attrition, accompanied by impaired synaptic plasticity (Fjell and Walhovd, 2010; Morrison and Baxter, 2012; Bartsch and Wulff, 2015; Fan et al., 2018; Mattson and Arumugam, 2018). A key driver of these changes is thought to be aging-associated neuroinflammation, which also appears to differentially impact specific vulnerable cell populations in the brain (Sparkman and Johnson, 2008; Simen et al., 2011; Mattson and Arumugam, 2018). Correlated with these changes is the compromised structural/functional integrity of mitochondria and impaired neuronal bioenergetics, cumulative buildup of dysfunctional proteins and an unfolded protein response, markers of endoplasmic reticulum (ER) stress, failure to effectively scavenge reactive oxygen species (ROS) and oxidative damage, overlaid on a baseline substratum of neuroinflammatory changes, namely a disrupted cytokine milieu and microglial activation (Wyss-Coray, 2016; Mattson and Arumugam, 2018; Webb and Sideris, 2020; Uddin et al., 2021). While these changes overlap and correlate with each other, the causal association between these events still remains unclear. However, several studies highlight that these changes, namely enhanced oxidative stress and neuroinflammatory states, accompanied by impaired DNA repair and mitochondrial dysfunction may play a vital role in driving the synaptic, structural and functional impairments associated with aging (Raz and Rodrigue, 2006; Dröge and Schipper, 2007; Paradies et al., 2011; Regnell et al., 2012; Green and Nolan, 2014; Pluvinage and Wyss-Coray, 2020). While these molecular and cellular changes are vital drivers of determining the aging trajectory, they are further impacted by both genetic background and life-course factors (Yuan et al., 2009; Kõks et al., 2016; Zannas, 2018; Marini et al., 2020; Ancelin et al., 2021). In this review, we focus on the vital life-course factor of early life experience, which can exert a long-lasting impact in determining an organism’s aging trajectory and health-span, in particular impacting the quality and nature of brain aging. A working hypothesis suggests that early adversity sets up an underlying “allostatic load” which impacts the physiology of normal aging creating fertile conditions that hasten and compromise the brain aging trajectory (Figure 1B; Danese and McEwen, 2012; Epel and Prather, 2018).

Given that most of the literature addressing the impact of early adversity on the brain aging trajectory is based on rodent models, it is worth considering a comparative scale of the equivalent age stages between rodents and humans. We have described in Figure 1B the distinct stages of perinatal, childhood, adolescence, adult, middle-aged and aged, as the major epochs of life. The perinatal stage encompasses the window of life from 23 weeks of gestation onward till about 2 years of human age. While it is challenging to draw direct parallels, the emergence of developmental milestones suggest that postnatal day 1–10 for rodents is equivalent to 23–40 weeks of human gestation, and postnatal day 10–21 is comparable to the window from birth till 3 years of human age. The childhood window comprising of 2–11 years of age for humans is thought to have an equivalence based on developmental indices to postnatal day 20–35 in rodents. The adolescent phase in humans (12–18 years) is thought to be comparable to postnatal day 35–49 in rodent models, with adulthood (20 years onward) compared to rodent models commencing from postnatal day 60 onward. The middle-aged and aged windows are generally thought to commence from 40 and 60 years of age respectively in humans, which has been suggested to compare to 9–15 months for middle-aged and 18 months upward as aged in rodent models (Flurkey et al., 2007; Semple et al., 2013; Dutta and Sengupta, 2016; Agoston, 2017; Wang et al., 2020).

## Early Adversity, Mitochondrial Dysfunction and Oxidative Stress

Mitochondria are an integrative hub that sense, adapt to and drive cellular stress responses, shaping the homeostatic adaptations to stress (Eisner et al., 2018; Picard et al., 2018). Mitochondria respond dynamically to stress signaling cues and mitokines, adjusting both architecture and function to rapidly adapt to altered energetic demands (Picard et al., 2015; Daniels et al., 2020). This ability of mitochondria to orchestrate effective cellular stress responses is a key component of the “resilient” phenotype (Hoffmann and Spengler, 2018), and a decline in this buffering capacity is linked to cellular senescence (Correia-Melo et al., 2016; Vasileiou et al., 2019). Early stress is speculated to deteriorate in the stress-buffering capacity of mitochondria, *via* a disrupted mitostasis, and thus accelerate senescence and neuronal damage, a cumulative consequence of brain aging (Figure 1C; Tyrka et al., 2016; Hoffmann and Spengler, 2018; Zitkovsky et al., 2021).

Studies using models of fragmented maternal care indicate both short (postnatal day 9) and long-term (10–12 months) changes in mitochondrial function within limbic brain regions and the periphery (Ruigrok et al., 2021). Adult progeny with a history of LBN exhibit perturbed electron transport chain (ETC) activity in the hypothalamus, and altered mitochondrial fusion/fission associated gene expression in the hippocampus upto 1 year of age (Ruigrok et al., 2021). MS evokes dysregulation of mitochondrial sirtuins within the PFC that persist well into middle-aged life (15 months) (Pusalkar et al., 2016), and robust decreases in mitochondrial mass in the periphery, namely the muscle, noted 8 months post the cessation of MS (Ghosh et al., 2016). Further, MS animals exhibit enhanced sensitivity to oxidative stress in peripheral mononuclear cells, noted until 18 months of age, and also reported in gut epithelial cells when examined in adulthood (2 months) in MS animals (Grigoruta et al., 2020; Khorjahani et al., 2020). Impaired calcium homeostasis, enhanced ROS and a decrease in oxygen consumption rate (OCR) or ATP production is also reported in the PFC, raphe and hippocampus of adult (2–6 month) MS animals, suggestive of a broad mitochondrial dysfunction in multiple systems (Della et al., 2013; Amini-Khoei et al., 2017; Masrour et al., 2018; Nold et al., 2019). Proteomic studies in diverse early stress models, spanning analysis from 12–24 weeks of age, indicate a dysregulation of proteins associated with mitochondrial energy metabolism in the PFC and hippocampus (Marais et al., 2009; Mairesse et al., 2012; Föcking et al., 2014; van Zyl et al., 2016; Nold et al., 2019), with a specific study suggesting a temporal variation in these effects noted at postnatal day 21 and 3–4 months following LBN, accompanied by a sex-specific differential expression of the hippocampal proteome at these timepoints (Eagleson et al., 2020). Furthermore, MS regulated glyoxalase enzymatic machinery at around 3 months of age that could result in a build-up of the pro-oxidant, methylglyoxal, which is a precursor of advanced glycation end-products implicated in neurodegeneration (Marais et al., 2009; Allaman et al., 2015). Early stress of MD also reduced superoxide dismutase and catalase activity in the hippocampus and PFC observed as early as postnatal day 20 and persisting into young adulthood (2 months), which could exacerbate oxidative stress in vulnerable limbic neurocircuits (Réus et al., 2017; Talukdar et al., 2020; Abelaira et al., 2021). Collectively, most reports of mitochondrial dysfunction following early stress restrict analysis to young adulthood (2–4 months of age) (Della et al., 2013; Masrour et al., 2018; Eagleson et al., 2020; Lapp et al., 2020), with few exceptions examining the consequences either in early postnatal life or well into middle-aged life ranging from 8–15 months (Ghosh et al., 2016; Pusalkar et al., 2016; Ruigrok et al., 2021). A careful analysis of the impact of early stress on the ontogeny of mitochondria within neuronal circuits, interaction with variables such as sex and genetic background remains to be extensively explored. Such studies are vital because a single-snapshot cannot capture the continuum of mitochondrial functional changes following early adversity, and it is likely that organ systems and brain regions will exhibit distinct timelines with different inflection points when adaptive attempts tip into maladaptive outcomes (Suri and Vaidya, 2015). Thus far the emerging picture raises the intriguing possibility that cumulative mitochondrial allostatic load following early adversity could sow the seeds for the hastening of age-associated impairments (Daniels et al., 2020).

## Early Adversity, Impaired Proteostasis and Autophagy

Early stress is speculated to alter proteostasis, trigger abnormal unfolded protein responses (UPR), and drive impaired autophagy thus establishing a substrate for aging-associated neuropathology (Figure 1C; Pinto et al., 2016; Liu et al., 2018; Criado-Marrero et al., 2019; Saulnier et al., 2021; Sierra-Fonseca et al., 2021). Maintaining effective protein quality is a multistep process spanning from synthesis, appropriate folding and conformational stability to turnover, and is vital in neurons that do not have the scope of cellular replacement to maintain the proteome (Muñoz-Carvajal and Sanhueza, 2020; Giandomenico et al., 2021; Saulnier et al., 2021). The proteostasis network, consisting of proteasome-dependent degradation machinery and autophagic processes is critical to maintaining the integrity of the functional proteome. Aging-dependent progressive decline in the efficiency of the proteostatic network is implicated in the establishment of neurodegeneration (Giandomenico et al., 2021). MS evokes significant disruption in expression of components of both the ubiquitin-proteasome system and the autophagy-lysosomal pathway in the hippocampus with changes noted in young adulthood (3 months), and specific alterations persisting well into middle-aged life (16 months). It is interesting that these changes appear to be restricted to the hippocampus and are not observed in the neocortex, suggesting differential vulnerability of neuronal circuits (Sierra-Fonseca et al., 2021). A recent report indicates that MIA evokes a sex-specific integrated stress response, evoking disrupted proteostasis in the cortex of embryonic 14.5 and 18.5 day old male fetuses, that is linked to the emergence of perturbed social and stereotypic behavior *via* a cytokine-dependent mechanism (Kalish et al., 2021). Early adversity could aggravate the aging-evoked UPR, in particular in the context of mitochondrial proteins, and serve as an early molecular signature that accelerates neuronal impairment (Muñoz-Carvajal and Sanhueza, 2020). Postnatal metabolic stress results in a perturbed UPR in the hippocampus and hypothalamus at 3 months of age, raising the intriguing possibility that the toxic combination of early adversity and metabolic insults could be a potent insult that disrupts healthy brain aging in animals as early as young adulthood (Pinto et al., 2016; Chen et al., 2021). Early adversity is a risk factor for neurodegeneration, which is linked to a disruption of proteostasis and perturbed amyloidogenic processing in 6–12 month old genetic mouse models of Alzheimer’s disease subjected to LBN (Sarter and Bruno, 2004; Dallé and Mabandla, 2018; Lesuis et al., 2018b,a). LBN enhances hippocampal Aβ40 and Aβ42 levels, primary components of amyloid plaques, in 6–12 months old male animals (Lesuis et al., 2018b). Further, gestational stress, MS and LBN enhance plaque burden, hasten cognitive decline noted at 9–12 months of age and shorten life expectancy in genetic mouse models of Alzheimer’s disease (Lesuis et al., 2016, 2018b; Hui et al., 2017; Jafari et al., 2019). However, there are also contradictory reports, wherein LBN does not alter the course of cognitive or neurogenic decline in 8–10 month old genetic Alzheimer’s disease animal models (Hoeijmakers et al., 2018). Whilst several reports link early adversity to mitochondrial dysfunction, there is still a paucity of detailed reports examining the influence of early adversity on proteostasis, UPR and autophagy in the brain, in particular across the life-span.

## Early Adversity, Epigenetic and Transcriptional Dysregulation

Amongst the foremost candidates for mediating the persistent effects of early adversity is a perturbed epigenetic landscape, thus driving transcriptional changes that hasten aging-evoked changes (Szyf, 2009; Doherty and Roth, 2018; Zannas, 2019; Palma-Gudiel et al., 2020). In animals exposed to early adversity, cognitive decline emerges as early as 12 months, and has been correlated with an altered epigenome in the hippocampus and PFC (Brunson et al., 2005; McClelland et al., 2011; Suri et al., 2013, 2014; Short et al., 2020). Several studies indicate altered expression of epigenetic machinery and of epigenetic modifications in the promoter regions of stress-responsive genes, such as the glucocorticoid receptor (GR) and brain derived neurotrophic factor (BDNF), with only a few reports examining these changes across the life-span (Roth and Sweatt, 2011; Suri et al., 2013; Pusalkar et al., 2016; Seo et al., 2016, 2020; Liu and Nusslock, 2018; Mourtzi et al., 2021). Several of the epigenetic and transcriptional changes evoked by early adversity are sex-dependent (Parel and Peña, 2022). MS is associated with dysregulated expression of the “writer” and “eraser” class of histone modifying enzymes, as well as DNA modifying enzymes, which in specific cases persist across the life-span, well into middle-aged life (15 months) (Pusalkar et al., 2016). This could contribute to global transcriptional changes in limbic brain regions, in particular within the hippocampus as observed at 15 months of age by Suri et al. (2014) (Marrocco et al., 2019; Peña et al., 2019; Usui et al., 2021). 15 month old MS animals exhibit perturbed expression of genes associated with calcium homeostasis, neuroinflammation, synaptogenesis, autophagy, proteasomal function, and cellular responses to stress (Suri et al., 2014). The nature of transcriptional dysregulation evoked by early adversity varies based on age, highlighting the importance of life-span studies (Suri et al., 2014). Amongst the key genes targeted by early adversity is GR, which plays a key role in mediating stress responses and HPA axis regulation. Diverse models of early adversity exhibit enhanced CpG methylation at the GR promoter in the hippocampus in young adults, driving reduced GR expression and disrupting the negative feedback regulation of the HPA axis (Weaver, 2007; Smart et al., 2015). This would enhance circulating corticosteroid levels, thus impacting neuronal atrophy and cognitive decline (McEwen, 2007). Aging is associated with enhanced circulating corticosteroid that negatively impact hippocampal neuron structure and function (Yau and Seckl, 2012). Following early adversity, animals have enhanced baseline, circadian and stress-evoked corticosteroid levels, compromising hippocampal cytoarchitecture/function and enhancing cognitive decline (McEwen, 2007). GRs and BDNF, both of which are dysregulated by early adversity, profoundly influence mitochondria (Daskalakis et al., 2015). GRs translocate into mitochondria, can regulate oxidative phosphorylation associated nuclear-encoded and mitochondrial gene expression, and influence bioenergetics (Psarra and Sekeris, 2009; Picard et al., 2014). BDNF, which is shown to exhibit a robust decline in the hippocampus and PFC of 15 month old animals subjected to early stress, can influence mitochondrial biogenesis and transport (Roth et al., 2009; Suri et al., 2013; Markham et al., 2014). The disrupted dyad of BDNF-GR signaling could influence cellular changes spanning from altered mitochondrial structure/function to dendritic atrophy, and at the organismal level perturb the neuroendocrine milieu and drive neurodegenerative decline (Rothman and Mattson, 2013; Suri and Vaidya, 2013; Daskalakis et al., 2015). A prior study indicates a marked reduction in expression of genes linked to antioxidant responses and DNA repair in the aging human neocortex after 40 years of age with enhanced oxidative DNA damage associated with the promoters of these downregulated genes likely due to attenuated base-excision repair mechanisms (Lu et al., 2004). Though speculative, one can envisage that early adversity could cumulatively enhance oxidative damage to DNA, RNA, proteins, and lipids (Karanikas et al., 2021). This is supported by evidence of hastened telomere attrition noted in 4–5 year old children that experienced childhood maltreatment, and phenocopied in animal models of early adversity (Drury et al., 2012; Price et al., 2013; Ridout et al., 2018). Amongst the implicated mediators of such DNA damage and telomere shortening following early adversity are the toxic cocktail of glucocorticoid-evoked oxidative stress, mitochondrial dysfunction, enhanced proton leak and neuroinflammation (Swaab et al., 2005; Casagrande et al., 2020).

## Early Adversity, Neuroinflammation, Structural and Cognitive Decline

Early stress triggers neuroimmune responses that drive prolonged, pathological and maladaptive neuroinflammation (Ganguly and Brenhouse, 2015; Nettis et al., 2020). Neuroinflammatory states evoked by early stress have been reviewed extensively, with evidence pointing to an induction of inflammatory cytokines, astrogliosis, and microglial activation (Ganguly and Brenhouse, 2015; Desplats et al., 2020). Most studies examine consequences of early adversity in postnatal or adult life, and do not address the long-term consequences on neuroinflammation (Delpech et al., 2016; Réus et al., 2019; Desplats et al., 2020; Dutcher et al., 2020; Reshetnikov et al., 2020; Kim et al., 2021). One of the reports indicates that MS increases microglial numbers/activation in 10 month old animals (Criado-Marrero et al., 2020), but few studies have actually followed animals with a history of early adversity across the life-span, to address the temporal and circuit-specific emergence of neuroinflammatory signatures (Ganguly and Brenhouse, 2015; Tay et al., 2018; Andersen, 2022). However, it is noteworthy that in some models of early stress (MIA), neuroinflammatory changes do not appear to contribute to synaptic atrophy and cognitive decline, with no changes reported in microglia or reactive astrocytes in 22 month old animals with a history of MIA (Giovanoli et al., 2015). Adult female, but not male, mice (2–3 months of age) with a life history of being subjected to fragmented maternal care showed deficits in reversal learning, suggesting a sex-specific influence of early adversity on cognition (Goodwill et al., 2018). This raises the possibility that while neuroinflammation is a consequence of early adversity, it remains poorly understood whether it is a causal contributor to accelerated aging-evoked neuronal and functional decline (Merz and Turner, 2021). It also highlights the critical importance of addressing potential sex differences in the pattern, onset and magnitude of neuroinflammatory changes evoked by early adversity, as neurohormones may exert a profound impact in modifying the trajectory of neuroinflammatory signatures (Ganguly and Brenhouse, 2015; González-Pardo et al., 2020).

The aging brain has several hallmark features, including atrophy of vulnerable neuronal populations and marked cognitive impairments (Fjell and Walhovd, 2010). Amongst the brain regions most extensively studied in this regard are the PFC and hippocampus, with dendritic atrophy, reduced spine density, decreased hippocampal neurogenesis, cellular shrinkage and volumetric loss being the key reported features (Figure 1C; McEwen and Morrison, 2013; Bartsch and Wulff, 2015). Several of these changes evoked by early adversity have been shown to be sex-dependent. In rats exposed to pre-pubertal stress, adult hippocampal neurogenesis is altered in adulthood in males, but not in females (Brydges et al., 2018). Further, in rats exposed to MS, females exhibit a more elaborate dendritic morphology and reduced thin spine density in infralimbic pyramidal neurons of the mPFC, which is not observed in male rats when examined at postnatal day 40 (Farrell et al., 2016). Several of these changes arise in a milieu associated with enhanced oxidative stress, mitochondrial dysfunction, disrupted proteostasis, neuroinflammatory signatures and an epigenetic milieu that drives reduced growth factor and enhanced inflammatory cytokine expression (Mattson and Arumugam, 2018). Early stress is associated with a long-lasting BDNF dysregulation in the hippocampus and/or PFC noted well into aged life reported at the age of 15 months (Suri et al., 2013) and 22 months (Giovanoli et al., 2015), along with a robust decline in hippocampal neurogenesis reported at the age of 10 months (Ruiz et al., 2018) and 15 months (Suri et al., 2013). The effects of early adversity on neurotransmitters and growth factors are suggested to be sex-dependent, which has been reviewed extensively by Perry et al. (2021). Neural stem cells shift to quiescence with aging, but continue to show similar proliferative capacity upon activation. Early stress is suggested to impair this proliferative capacity in aging neural stem cells, dampening the capacity for repair (Suri et al., 2013; Kalamakis et al., 2019). Several early stress models (MS, LBN, MIA, and MD) exhibit significant cognitive impairments in middle-aged and aged life (Brunson et al., 2005; Sterlemann et al., 2010; Suri et al., 2013; Sousa et al., 2014; Giovanoli et al., 2015; Yajima et al., 2018). The preponderance of literature using early stress models reporting changes at the epigenetic, transcriptional, mitochondrial, proteostatic, neuroinflammatory, cytoarchitectural and behavioral level is at best correlative, but thus far does not provide a clear causal relationship between changes at distinct levels of organization that mechanistically drive the accelerated aging phenotype evoked by early adversity.

## Conclusion

Early adversity disrupts the functioning of key physiological processes that facilitate adaptive stress responses, setting in motion a cumulative “allostatic load” that alters the nature and time-line of healthy brain aging. Further, early adversity could also impact key neurodevelopmental milestones, which could alter the optimal functioning of neurocircuits thus setting up a substratum for a disruption of the trajectory for brain aging. The interim duration between exposure to early stressors and eventual brain aging outcomes provides a substantial temporal window for interventional approaches, including life-course factors such as exercise, diet, environmental enrichment, epigenetic and pharmacological interventions that may serve to reverse or ameliorate the negative impacts of early adversity on brain aging. Here we have provided an overview of the key brain aging-associated processes targeted by early adversity, and highlighted gaps in knowledge that require future investigation.

## Author Contributions

PC, AS, and VV jointly wrote the manuscript. All authors contributed to the article and approved the submitted version.

## Conflict of Interest

The authors declare that the research was conducted in the absence of any commercial or financial relationships that could be construed as a potential conflict of interest.

## Publisher’s Note

All claims expressed in this article are solely those of the authors and do not necessarily represent those of their affiliated organizations, or those of the publisher, the editors and the reviewers. Any product that may be evaluated in this article, or claim that may be made by its manufacturer, is not guaranteed or endorsed by the publisher.

## Abbreviations

MIA: maternal immune activation
LGABN: licking, grooming, arched back nursing
MD: maternal deprivation
MS: maternal separation
MSUS: maternal separation and unpredictable stress
LBN: limited bedding and nesting
PFC: prefrontal cortex
DNA: Deoxyribonucleic acid
ETC: electron transport chain
ROS: reactive oxygen species
OCR: oxygen consumption rate
ATP: Adenosine triphosphate
UPR: unfolded protein response
GR: glucocorticoid receptor
BDNF: brain derived neurotrophic factor
HPA: Hypothalamo-pituitary-adrenal axis
RNA: Ribonucleic acid.
